# A systematic review on chlorine dioxide as a disinfectant

**DOI:** 10.25122/jml-2021-0180

**Published:** 2022-03

**Authors:** Umi Haida Nadia Mohamed Jefri, Abdullah Khan, Ya Chee Lim, Kah Seng Lee, Kai Bin Liew, Yaman Walid Kassab, Chee-Yan Choo, Yaser Mohammed Al-Worafi, Long Chiau Ming, Anandarajagopal Kalusalingam

**Affiliations:** 1. PAP Rashidah Sa'adatul Bolkiah Institute of Health Sciences, Universiti Brunei Darussalam, Gadong, Brunei Darussalam; 2. School of Pharmacy, KPJ Healthcare University College, Nilai, Malaysia; 3. Faculty of Pharmacy, University of Cyberjaya, Cyberjaya, Malaysia; 4. College of Pharmacy, National University of Science and Technology, Muscat, Oman; 5. Faculty of Pharmacy, Universiti Teknologi MARA, Puncak Alam, Malaysia; 6. College of Pharmacy, University of Science and Technology of Fujairah, Fujairah, United Arab Emirates; 7. College of Medical Sciences, Azal University for Human Development, Sana'a, Yemen

**Keywords:** COVID-19, Chlorine dioxide, disinfectant, safety, efficacy

## Abstract

The COVID-19 pandemic has tremendously increased the production and sales of disinfectants. This study aimed to systematically review and analyze the efficacy and safety of chlorine dioxide as a disinfectant. The literature relating to the use of chlorine dioxide as a disinfectant was systematically reviewed in January 2021 using databases such as PubMed, Science Direct, and Google Scholar. Inclusion criteria were studies that investigated the use of chlorine dioxide to assess the efficacy, safety, and impact of chlorine dioxide as a disinfectant. Out of the 33 included studies, 14 studies focused on the disinfectant efficacy of chlorine dioxide, 8 studies expounded on the safety and toxicity in humans and animals, and 15 studies discussed the impact, such as water treatment disinfection using chlorine dioxide. Chlorine dioxide is a safe and effective disinfectant, even at concentrations as low as 20 to 30 mg/L. Moreover, the efficacy of chlorine dioxide is mostly independent of pH. Chlorine dioxide can be effectively used to disinfect drinking water without much alteration of palatability and can also be used to destroy pathogenic microbes, including viruses, bacteria, and fungi from vegetables and fruits. Our review confirms that chlorine dioxide is effective against the resistant *Mycobacterium*, H1N1, and other influenza viruses. Studies generally support the use of chlorine dioxide as a disinfectant. The concentration deemed safe for usage still needs to be determined on a case-by-case basis.

## INTRODUCTION

Disinfectants are used to eliminate organisms, either in chemical or physical forms. Disinfectants are usually applied to contaminated surfaces and are typically present as chemicals, soaps, or detergents [1]. Disinfectants act on micro-organisms by inhibiting the growth of micro-organisms, such as bacteria and fungi, or by having lethal action on viruses [2]. The current COVID-19 pandemic has enormously increased the production and sales of various disinfectants [3]. Different chemical structures with properties of disinfectants have been identified. These chemical structures include alcohol, aldehydes, anilides, biguanides, bisphenols, diamidines, halogen-releasing agents, halophenols, heavy metal derivatives, peroxygens, quaternary ammonium compounds, phenols, and cresols [4]. These chemicals are present in different disinfectants used for antisepsis, disinfection, preservation, sterilization, antiplaque agents' deodorants, and cleaning products. However, each disinfectant attacks different target areas of the micro-organisms. Certain disinfectants may not be effective in eliminating viruses as it depends on the nature of the disinfectants [4]. Disinfectants can be divided into two broad groups: oxidizing and non-oxidizing disinfectants. Disinfectants containing halogens such as chlorine, iodine, and oxygen releasing materials are called oxidizing disinfectants, while disinfectants that bond to structures such as quaternary ammonium compounds and amphoterics are known as non-oxidizing disinfectants [5].

The use of disinfectants in the water distribution system is also essential nowadays. The rationale for disinfecting water is to remove or inactivate micro-organisms present in raw water. It is also important to disinfect drinking water to prevent contamination and maintain the quality of drinking water throughout the distribution system [6]. Failure to disinfect drinking water properly may lead to water-borne infections caused by various bacteria, viruses, protozoa, and parasitic helminths. The risk of illness depends on the number of organisms ingested. Therefore, water disinfection is important to reduce water contamination and improve the health status of a country's population [7]. Chlorine-based disinfectants are primarily used in water treatment plants and play an important role in providing microbial-safe drinking water [6].

This review is focused on the strength and efficacy of disinfectants, specifically chlorine dioxide, in killing bacteria and other microbes. Chlorine dioxide was initially discovered in 1814 by Sir Humphrey Davy and was commercially produced in 1940 as a bleaching agent [8]. Currently, it is often used as a bleaching agent and water disinfectant. It is labeled as a strong oxidizing agent, microbicide, and antiseptic [9]. Furthermore, the safety of disinfectants for human use is also important to avoid any health-related problems. Therefore, this study aims to review and analyze the efficacy, safety, and impact of using chlorine dioxide as a disinfectant. The secondary aim of this review is to obtain information regarding the effective strength or concentration of chlorine dioxide. Another aim of this review is to evaluate the significance of chlorine dioxide in drinking water and wastewater as disinfectants and assess whether chlorine dioxide as a disinfectant effectively controls the growth of micro-organisms present in the respective water source. This systematic review is unique because it focuses on the general applications of chlorine dioxide and explores the safety, efficacy, and conventional and extended uses of this disinfectant.

## MATERIAL AND METHODS

### Search strategy

A comprehensive search was performed using PubMed, Science Direct, and Google Scholar (Table 1). This online search was done from the inception date until 2 January 2021. The following keywords were combined using the Boolean operators ("AND" & "OR”): Disinfectant, Disinfection, Chlorine dioxide, Strength, Effective, Efficacy, Safety, Toxicity Water, Wastewater, Drinking water.

**Table 1.** Summary of the database search.

Databases	Search Terms	Results
**Pubmed**	Disinfect* AND Chlorine dioxide AND Strength OR Effective OR Efficacy OR Effica* OR Safety OR Safe* OR Toxicity OR Water OR Waste water OR Drinking water.	741
**Science Direct**	Disinfectant OR Disinfection OR Disinfect* AND Chlorine dioxide AND Strength OR Effective OR Efficacy OR Effica* OR Safety OR Safe* OR Toxicity OR Water OR Waste water OR Drinking water.	792
**Google Scholar**	Disinfectant OR Disinfection OR Disinfect* AND Chlorine dioxide AND Strength OR Effective OR Efficacy OR Effica* OR Safety OR Safe* OR Toxicity OR Water OR Waste water OR Drinking water.	81

### Study selection

After removing duplicates, the titles and abstracts of the remaining studies were screened to identify relevant studies. In cases where insufficient information was available to identify a study's suitability from its title and abstract, the full text was screened to clarify eligibility. One reviewer (NMJ) independently evaluated the inclusion of each article; this was checked by a second reviewer (LCM), and any disagreement regarding the inclusion was resolved through extensive discussion with the third reviewer (YCL). Following an initial screening, the full text of the studies identified as potentially suitable for inclusion was reviewed. Studies were included if they had information regarding chlorine dioxide as a disinfectant, in English language and published in the last 40 years. Conference abstracts, blogs, review articles, and articles with less significance on the objective of this review were excluded from this review.

### Data Extraction

Based on the inclusion criteria, the articles were selected from the databases as mentioned in Table 1, a summary of the database search. The focus was on the titles describing the use of chlorine dioxide as a disinfectant, its safety, and its efficacy in decontaminating or preserving the products from contamination. Articles principally evaluating the efficacy of chlorine dioxide and potentially harmful (common) pathogens were mainly used.

## RESULTS

**Figure 1.**
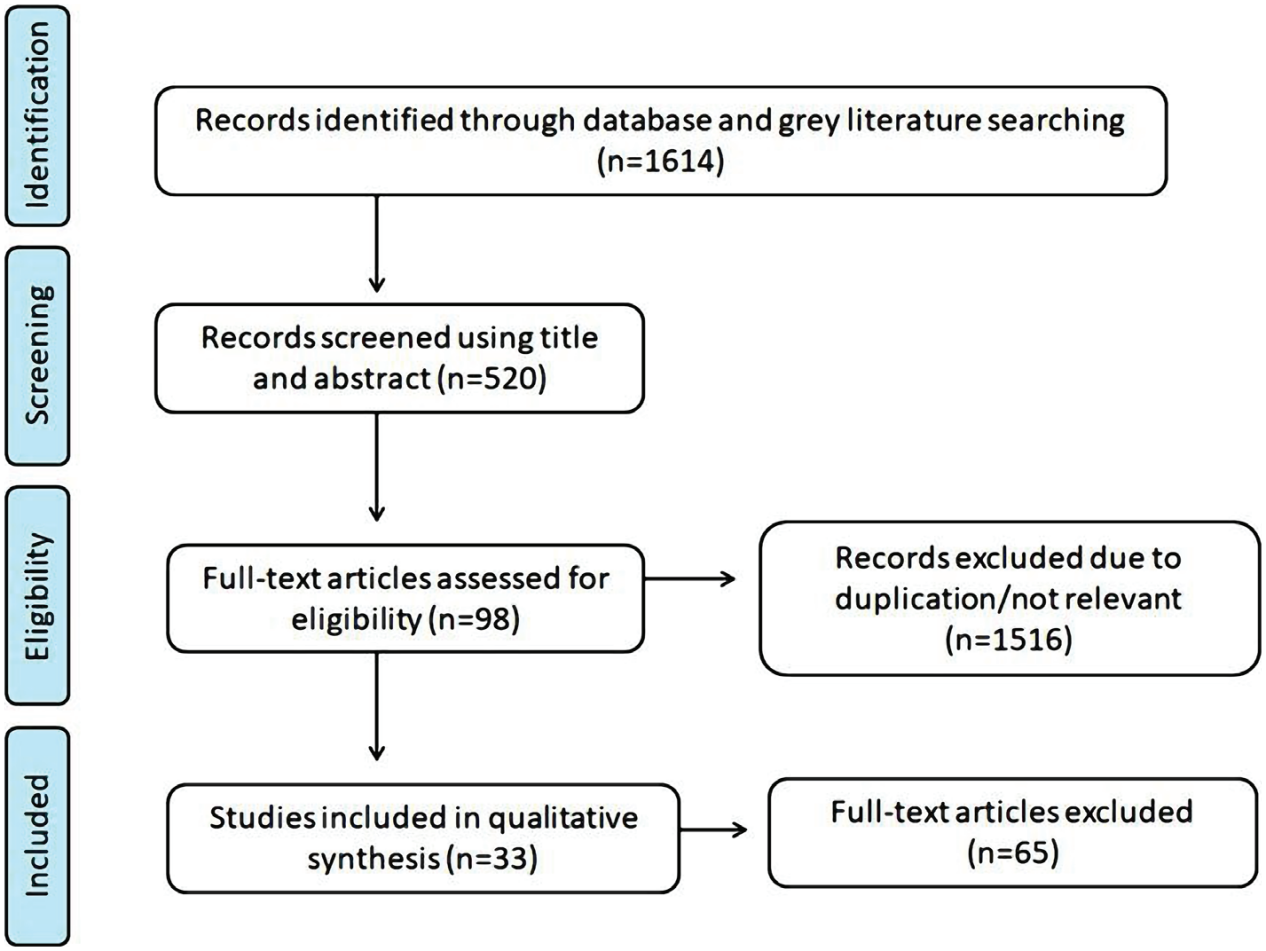
PRISMA flow chart.

A flow chart of the literature review search and identification of relevant papers is shown in Figure 1. In total, 1614 articles were obtained from all databases. Only 520 articles remained after duplicates were removed. Based on the inclusion and exclusion criteria, only about 98 articles were eligible for this review. Only 33 articles were included in the review for the final full-text assessment.

A total of 14 articles focused on different effective concentrations of chlorine dioxide against targeted micro-organisms. Furthermore, 8 articles focused on the safety and toxicity of chlorine dioxide in humans and animals. 4 out of 8 articles are included in other sections, where 3 articles also focused on the efficacy of chlorine dioxide and 1 on the impact of chlorine dioxide on water disinfection. The remaining 15 articles focused on disinfection of water, in which some focussed on the use and impact of water disinfection for both wastewater and drinking water. 8 out of 15 articles emphasized water disinfection for water in general.

References from studies included were screened to identify additional relevant studies. A detailed description of the results obtained is provided in Supplementary information 1: Efficacy of chlorine dioxide; Supplementary information 2: Safety of chlorine dioxide and Supplementary information 3: Impact of chlorine dioxide on water (drinking water and wastewater).

## DISCUSSION

### Disinfection efficacy using chlorine dioxide

The articles obtained for this section were published from 1998 to 2019. The most recent article evaluated the effectiveness of chlorine dioxide as a sanitizer on mixed salad to reduce the presence of human norovirus [10]. The study evaluated the efficacy of a chlorine dioxide solution prepared using 2000 mg/L of chlorine gas dissolved in water. This chlorine dioxide solution was tested against various bacteria and found to be effective in 5–20 mg/L with a 98.2% reduction in bacterial activity, whereas the antiviral effects against H1NI and EV71 strains were observed at 46.39 mg/L and 84.65 mg/L, respectively. The prepared chlorine dioxide solution was safe when tested in rabbits at 50 mg/L concentration. Moreover, 40 mg/L in drinking water did not show any toxic symptoms when tested for subchronic oral toxicity. The most similar article to this review was published by Ma *et al.*, which evaluates the efficacy and safety of chlorine dioxide solution [11]. Chlorine dioxide was effective against the virus strain at 20 mg/L, reducing virus infectivity by at least 3-log units after 1 min. Other studies focused on the efficacy of oxidizing disinfectants such as chlorine dioxide to inactivate murine norovirus [12] and on the inactivation of bacteria and viruses when a low concentration of chlorine dioxide gas was used [13].

Three articles obtained from 2013 were published by Hsu and Huang (2013), Watamoto *et al.* (2013), and Xue *et al.* (2013) [14–16]. Hsu and Huang (2013) focused on the disinfection efficacy of chlorine dioxide gas [14]. The study found that the one-time disinfection process reduced the fungal and bacterial loads by 30% and 65%, respectively. Spraying disinfectant twice a day reduced the bacterial load by 74% and the fungal load by 38%. Watamoto *et al.* reported that disinfection of dental instruments using 0.02% chlorine dioxide completely disinfected the instrument within 10 minutes of application [15]. The chlorine dioxide solution at 0.02% concentration even removed HCV from the contaminated instruments. Thus, the authors suggested that chlorine dioxide and ultrasonic disinfectants provide a suitable alternative to other potentially toxic disinfectants. Moreover, Xue *et al.* (2013) informed the effects of chlorine dioxide on human rotavirus [16]. They found chlorine dioxide to be more effective than chlorine alone. The efficiency of disinfection is defined using the Ct value as the product of disinfectant concentration (milligram per liter) and the contact time (min). A Ct value of chlorine dioxide and chlorine of 1.21–2.47 mg/L and 5.55–5.59 min, respectively, were required at 20°C for a 4-log reduction of HRV.

Furthermore, Miura and Shibata (2010) described the efficacy of chlorine dioxide against the influenza virus [17]. They suggested that chlorine dioxide has strong disinfectant activity at a concentration of 0.03%. Their review suggested that chlorine dioxide, either in a solution or gaseous form, could be effectively used to control influenza due to its strong antiviral effects used to control H1N1 infections. Other articles that show the efficacy of chlorine dioxide on bacteria were published by Valderrama *et al.*, Taylor *et al.* and Foschino *et al.*, where Valderrama *et al.* reported a 4-log unit reduction in the Listeria monocytogenes upon treatment with 3 mg/L chlorine dioxide within 90 seconds [18–20]. However, the efficacy of chlorine dioxide was not the same for 20% CaCl2 brine solutions; this inefficacy could be due to the interference or presence of the divalent cations and organic materials. Taylor *et al.* showed disinfecting properties of disinfectants on *Mycobacterium avium*, and Foschino *et al.* illustrated its killing effects on *Escherichia coli* [19, 20]. Zhu *et al.* distinctly narrated the preparation of solid chlorine dioxide-based disinfectant powder and its efficacy [21]. This disinfectant powder was diluted with water before use and tested against *Staphylococcus aureus* and *Escherichia coli*. The study concluded that vegetative forms of bacteria could be killed using 100 mg/L chlorine dioxide solution. Moreover, the same concentration of chlorine dioxide effectively disinfected the surface objects. The powder was found to be safe and non-irritant.

Gagnon *et al.* published an article in 2005 showing the efficacy of chlorine dioxide in drinking water biofilms [22]. Their study revealed that chlorine dioxide (0.5 mg/L) has greater efficacy against heterotrophic bacteria than chlorite ions. The last article to be mentioned was published in 2003 by Jean *et al.*, focusing on the effectiveness of commercial disinfectants, including chlorine dioxide, to inactivate the Hepatitis A virus [23]. They investigated six commercially available disinfectants, including 2% stabilized chlorine dioxide solution against Hepatitis A virus in suspensions or on the food and other surfaces. Disinfectants were more effective in suspensions compared to their efficacy against the virus on food or other surfaces. Chlorine dioxide 30 mg/L demonstrated a 0.96 and 1.01 viral reduction (log) at 4°C and 22°C, respectively.

### Safety and toxicity of chlorine dioxide disinfectant on humans and animals

The articles obtained were published from 1983 to 2019. Eight articles described the safety and toxicity of chlorine dioxide disinfectants, with 4 of them including other subtopics. Three out of 4 articles were obtained from the first subtopic, the efficacy of chlorine dioxide, published by Ma *et al.*, Miura and Shibata, and Zhu *et al.* in 2017, 2010, and 2008, respectively [11, 17, 21]. The other article was obtained from the third and final subtopics of this review, where it was added later on after full-text review, and it was published by Zhong *et al.* in 2019 [24]. The remaining 4 articles focus on the safety and toxicity of chlorine dioxide specifically. Two of the articles published in 2005 by Ferraris *et al.* and Svecevicius *et al.* explored the toxicity of chlorine dioxide on rainbow trout [25, 26]. Ferraris *et al.* utilized the hepatocytes of the rainbow trout fish, while Svecevicius *et al.* used the fish as a whole for the study [25, 26]. Peter Bercz *et al.* published an article in 1986 showing the toxicity of chlorine dioxide on iodide metabolism, which is part of the thyroid function in humans, and animals were used in this study [27]. Another article was published in 1983 by Duck H. Suh, showing the toxicity of chlorine dioxide present in drinking water on fetal development in rats [28].

### Impact of water disinfection using chlorine dioxide

Fifteen articles discussed the impact of water disinfection using chlorine dioxide, with 3 focusing on wastewater disinfection, 4 detailing drinking water disinfection, and 8 informing on water disinfection in general. The articles obtained for wastewater disinfection were published by Zhong *et al.*, Akhlaghi *et al.*, and Alcalde *et al.* in 2019, 2018, and 2007, respectively [24, 29, 30]. Zhong *et al.* explained disinfection by-products (DBPs) in wastewater effluents treated by chlorine dioxide [24]. Akhlaghi *et al.* compared data of chlorine dioxide and chlorine disinfectant in wastewater effluent, while Alcalde *et al.* gave information on wastewater reclamation systems that use chlorine dioxide to disinfect water [29, 30]. The articles for drinking water disinfection were published by Lin *et al.*, Sorlini *et al.*, Lenes *et al.*, and Wondergem and van Dijk-Looijard in 2014, 2010, and 1991, [31–34]. Lin *et al.* and Sorlini *et al.* published in 2014, and Lin *et al.* studied the use of chlorine dioxide to inactivate zooplankton in drinking water treatment. Sorlini *et al.* indicated the influence of drinking water treatment on chlorine dioxide consumption [31, 32].

Lenes *et al.* assessed the removal and inactivation of influenza viruses using a drinking water treatment, while Wondergem and van Dijk-Looijard evaluated the use of chlorine dioxide as a post-disinfectant for drinking water [33, 34]. The remaining eight articles reported on water disinfection in general. These eight articles were published by Wen *et al.*, Casini *et al.*, Qiao *et al.*, Heiner *et al.*, Liu and Lin, Barbeau *et al.*, Chauret *et al.*, and Huber *et al.* for water disinfection in general [35–42]. Wen *et al.* evaluated the inactivation of fungal spores in groundwater disinfected using chlorine dioxide [35]. Casini *et al.* provided information on the long-term effect of hospital water network disinfection on Legionella and waterborne bacteria [36]. Qiao *et al.* evaluated the inactivation of resistant *Mycobacterium mucogenicum* in water due to chlorine resistance [37]. Heiner *et al.* compared a variety of field water disinfection tablets [38]. Liu and Lin studied the application of chlorine dioxide for water disinfection [39]. Barbeau *et al.* showed the impact of water quality in natural waters [40]. Chauret *et al.* showed the effect of disinfectants on micro-organisms in the water distribution systems, and Huber *et al.* illustrated the potential oxidation of chlorine dioxide during water treatment [41, 42].

A few of the articles may provide both qualitative and quantitative data. However, most articles focus on qualitative data only. Most of the studies were done in a laboratory setting, in a controlled manner. Not all of the studied articles provided information on chlorine dioxide alone. Most studies also include other disinfectants where they compare the differences between each disinfectant. Even if the articles include other disinfectants, chlorine dioxide is the main disinfectant to be reviewed, and only the data including chlorine dioxide was compiled and compared in the different settings from each article. Additional data in the articles with less significance for this review were excluded.

### Efficacy of chlorine dioxide

In general, the studied articles gathered came to the same conclusion that chlorine dioxide is effective against micro-organisms tested [10–18, 20–23]. Not all of the concentrations used in all of the studies were the same; therefore, it is better to evaluate whether or not chlorine had its effect on different types of micro-organisms. The only possible reason for using different concentrations of chlorine dioxide may be based on the guidelines on the micro-organisms tested. As a result, most of the studies that tested the efficacy of chlorine dioxide against viruses used a higher concentration of chlorine dioxide compared to the concentration used for bacteria and fungi [10–12, 15, 17, 23]. For bacteria and fungi, even the lowest possible concentration of chlorine dioxide gives an effect. As a result, even the lowest concentration used shows a certain level of its effect, and the highest concentration gives a complete removal of micro-organisms [11, 13, 23].

When chlorine dioxide is compared with other disinfectants, it is better to keep in mind that most studies used a lower concentration of chlorine dioxide than other disinfectants [10, 16, 22, 23]. Even at a low concentration of chlorine dioxide used, it is effective against the tested micro-organisms. Another disinfectant, such as hypochlorite and peracetic acid, was used at a higher concentration which may be 2 to 3 times higher and have a high efficacy [10]. Therefore, using chlorine dioxide lower than that and still being effective was considered more effective.

However, there are also some anomalies in the included studies regarding the efficacy of chlorine dioxide. For example, one study mentioned that chlorine dioxide does not have an effect as there was no significant difference before and after disinfection [22]. Another anomaly is that the use of chlorine dioxide may lead to resistance, as there was evidence on *Mycobacterium avium strains* developing resistance to chlorine dioxide [19].

### Chlorine dioxide toxicity in humans and animals

Chlorine dioxide is relatively safe for use based on the studies. The possibility of chlorine dioxide being cytotoxic to humans is low [11]. Each study states the maximum concentration of chlorine dioxide that is safe for use, and usually, levels beyond the maximum concentration of chlorine dioxide may show some cytotoxicity. The maximum concentration of chlorine dioxide differs from one animal to another. The chlorine dioxide concentrations that may become toxic are size or weight-related. The reason for this hypothesis was that one of the animals, the African monkeys, can tolerate a higher concentration of chlorine dioxide compared to rats, which are smaller in terms of physical size [17].

When chlorine dioxide was compared with another disinfectant such as chlorine, it was proven that both disinfectants were safe for use. However, when chlorine and chlorine dioxide were mixed, it had a higher cytotoxicity level, especially on fish [24]. Therefore, using chlorine and chlorine dioxide is possible, but advisable to avoid using them as a disinfectant in water containing aquatic animals. Disinfectant by-products (DBPs) released by chlorine dioxide have a higher toxicity level compared to chlorine dioxide based on the results. It is worth noting that between chlorite and chlorate, chlorate has a higher possibility of being toxic [24, 28]. Therefore, it is advisable to avoid using chlorine dioxide disinfectant that is less stable to reduce the production of disinfectant by-products (DBPs).

### Water disinfection using chlorine dioxide

Not all studies suggest that using chlorine dioxide has a positive impact on water disinfection. Three studies on chlorine dioxide on wastewater effluents generally reported a positive impact. This positive impact was based on the importance of chlorine dioxide in the water treatment of wastewater as using only chlorine as a disinfectant for water treatment is insufficient [24, 29, 35, 39, 42]. This supports the use of chlorine dioxide owing to its higher efficacy compared to chlorine. One of the studies suggested that chlorine alone may increase the production of disinfectant by-products (DBPs). When chlorine was used together with chlorine dioxide, the production of these disinfectant by-products (DBPs) was much lower and less likely to have a toxic effect [24].

In four studies on the use of chlorine dioxide in drinking water, chlorine dioxide also had a positive impact on drinking water disinfection [31, 32, 34, 38]. The studies indicate that chlorine dioxide used for drinking water treatment is beneficial. The study also supports chlorine dioxide in the water treatment with the additional effect of inactivating influenza viruses such as H5N1 and H1N1 and other disinfectants to allow full water disinfection against harmful micro-organisms [33].

In addition, these studies also provide alternatives for water disinfection plants to increase the efficacy of chlorine dioxide for drinking water treatment [31, 32]. To make full use of chlorine dioxide as a disinfectant for drinking water, some chemicals should be avoided or could be added. The chemical that should be avoided with chlorine dioxide in water treatment is an oxidizing organic matter as this may lead to the lower inactivation rate of micro-organisms present in drinking water. This will lead to a possible presence of micro-organisms in the drinking water. In addition, using aluminum sulfate to replace iron (III) chloride may be used in some water plants [32]. The reason for this replacement is to reduce the concentration of chlorine dioxide used for water treatment.

The remaining studies highlighting water disinfection in general also proved that chlorine dioxide in water treatment plants had a positive impact. As mentioned by one of the studies, the inactivation rate of fungal spores was increased when chlorine dioxide was used, suggesting a positive impact on water treatment [35]. Similar to the result of using chlorine dioxide in drinking water, chlorine dioxide is more efficient than chlorine as a water disinfectant. One of the articles also highlights that chlorine dioxide only acts as a partial barrier, especially in the case of pharmaceutical oxidation reactions [42]. Other information regarding the use of chlorine dioxide for water disinfection that deviates from the general result of the studies includes the resistance of micro-organisms, palatability of water, and insufficient evidence on the impact of using chlorine dioxide. The use of chlorine dioxide and possibly a longer time frame of using chlorine dioxide may lead to resistance. This was proven by two studies, with evidence on specified micro-organisms that were more likely to develop resistance against chlorine dioxide [35, 37]. As chlorine dioxide derives from chlorine, there is a possibility of developing resistance to chlorine dioxide, for example, mycobacteria. As mentioned by the study, the compositions of mycobacteria cell membrane might lead to chlorine resistance [37]. Therefore, it may also affect chlorine dioxide resistance. The palatability of water is not preferred when chlorine dioxide is used alone. To improve this palatability of water, an additional reagent such as ascorbic acid would help. Even with the addition of ascorbic acid, the most preferred one is not chlorine dioxide and ascorbic acid. However, when comparing using chlorine dioxide alone and using chlorine dioxide with ascorbic acid, it is preferable to add ascorbic acid [38].

The current best disinfectant used for drinking water was chlorine dioxide compared to chlorine. Moreover, the method of water disinfection is also important to ensure the availability of palatable water to the target population. Especially for smaller areas or countries where the budget is limited, some methods such as the infiltration-percolation system could be more efficient in cost saving. However, this water disinfection method should be monitored and adjusted from time to time in the long-term run of using chlorine dioxide to ensure that there is enough evidence to fully support the use of chlorine dioxide.

## CONCLUSIONS

This review analyzed the efficacy, safety, and influence of chlorine dioxide on water disinfection. The referred literature supports chlorine dioxide as a safe and efficient disinfectant. Generally, chlorine dioxide is recommended as a disinfectant since it kills microorganisms even at low concentrations. Chlorine dioxide could be used as a disinfectant during the COVID-19 pandemic. Studies also support the use of chlorine dioxide in water treatment, especially in delivering microbial-safe drinking water. The use of chlorine dioxide is a newer water disinfection technology; adequate management of the method is essential to make it cost-effective and ensure high efficacy and safety for humans and animals.

## ACKNOWLEDGMENTS

### Conflict of interest

The authors declare no conflict of interest.

### Authorship

LCM contributed to conceptualizing; UHNMJ, YMA, LCM, AK contributed to the methodology; UHNMJ, AK, YCL, LCM, AK contributed to writing the original draft; UHNMJ, AK, LCM, AK contributed to editing the manuscript; UHNMJ, YWK, CC contributed to data collection; UHNMJ, KSL, KBL contributed to data curation.

## References

[1] Kahrs RF (1995). General disinfection guidelines. Rev Sci Tech.

[2] Maris P (1995). Modes of action of disinfectants. Rev Sci Tech.

[3] Boyce JM (2016). Modern technologies for improving cleaning and disinfection of environmental surfaces in hospitals. Antimicrobial Resistance & Infection Control.

[4] Skelley J (2016). Open source tactics: Bargaining power for strategic litigation. J Intellect Prop.

[5] Fisher J (2003). Cleaning procedures in the factory - Types of Disinfectant. Encycl Food Sci Nutr.

[6] Whitacre DM (2013). Preface. Reviews of environmental contamination and toxicology.

[7] Backer HD, Sanford CA, Pottinger PS, Jong EC (2017). Chapter 7 - Water Disinfection. The Travel and Tropical Medicine Manual.

[8] Gray NF, Percival SL, Yates MV, Williams DW, Chalmers RM, Gray NF (2014). Chapter Thirty-Two - Chlorine Dioxide. Microbiology of Waterborne Diseases.

[9] Gómez-López VM, Wexler P (2014). Chlorine Dioxide. Encyclopedia of Toxicology.

[10] Anfruns-Estrada E, Bottaro M, Pintó RM, Guix S, Bosch A (2019). Effectiveness of Consumers Washing with Sanitizers to Reduce Human Norovirus on Mixed Salad. Foods.

[11] Ma JW, Huang BS, Hsu CW, Peng CW (2017). Efficacy and Safety Evaluation of a Chlorine Dioxide Solution. Int J Environ Res Public Health.

[12] Girard M, Mattison K, Fliss I, Jean J (2016). Efficacy of oxidizing disinfectants at inactivating murine norovirus on ready-to-eat foods. International Journal of Food Microbiology.

[13] Ogata N, Sakasegawa M, Miura T, Shibata T (2016). Inactivation of Airborne Bacteria and Viruses Using Extremely Low Concentrations of Chlorine Dioxide Gas. Pharmacology.

[14] Hsu CS, Huang DJ (2013). Disinfection efficiency of chlorine dioxide gas in student cafeterias in Taiwan. J Air Waste Manag Assoc.

[15] Watamoto T, Egusa H, Sawase T, Yatani H (2013). Clinical evaluation of chlorine dioxide for disinfection of dental instruments. Int J Prosthodont.

[16] Xue B, Jin M, Yang D, Guo X (2013). Effects of chlorine and chlorine dioxide on human rotavirus infectivity and genome stability. Water Res.

[17] Miura T, Shibata T (2010). Antiviral Effect of Chlorine Dioxide against Influenza Virus and Its Application for Infection Control. The Open Antimicrobial Agents Journal.

[18] Valderrama WB, Mills EW, Cutter CN (2009). Efficacy of chlorine dioxide against Listeria monocytogenes in brine chilling solutions. J Food Prot.

[19] Taylor RH, Falkinham JO, Norton CD, LeChevallier MW (2000). Chlorine, chloramine, chlorine dioxide, and ozone susceptibility of *Mycobacterium avium*. Appl Environ Microbiol.

[20] Foschino R, Nervegna I, Motta A, Galli A (1998). Bactericidal activity of chlorine dioxide against *Escherichia coli* in water and on hard surfaces. J Food Prot.

[21] Zhu M, Zhang LS, Pei XF, Xu X (2008). Preparation and evaluation of novel solid chlorine dioxide-based disinfectant powder in single-pack. Biomed Environ Sci.

[22] Gagnon GA, Rand JL, O'leary KC, Rygel AC (2005). Disinfectant efficacy of chlorite and chlorine dioxide in drinking water biofilms. Water Res.

[23] Jean J, Vachon JF, Moroni O, Darveau A (2003). Effectiveness of commercial disinfectants for inactivating hepatitis A virus on agri-food surfaces. J Food Prot.

[24] Zhong Y, Gan W, Du Y, Huang H (2019). Disinfection byproducts and their toxicity in wastewater effluents treated by the mixing oxidant of ClO2/Cl2. Water Research.

[25] Ferraris M, Chiesara E, Radice S, Giovara A (2005). Study of potential toxic effects on rainbow trout hepatocytes of surface water treated with chlorine or alternative disinfectants. Chemosphere.

[26] Svecevicius G, Syvokiene J, Stasiŭnaite P, Mickeniene L (2005). Acute and chronic toxicity of chlorine dioxide (ClO2) and chlorite (ClO2-) to rainbow trout (Oncorhynchus mykiss). Environ Sci Pollut Res Int.

[27] Bercz JP, Jones LL, Harrington RM, Bawa R, Condie L (1986). Mechanistic aspects of ingested chlorine dioxide on thyroid function: impact of oxidants on iodide metabolism. Environ Health Perspect.

[28] Suh DH, Abdel-Rahman MS, Bull RJ (1983). Effect of chlorine dioxide and its metabolites in drinking water on fetal development in rats. J Appl Toxicol.

[29] Akhlaghi M, Dorost A, Karimyan K, Narooie MR, Sharafi H (2018). Data for comparison of chlorine dioxide and chlorine disinfection power in a real dairy wastewater effluent. Data Brief.

[30] Alcalde L, Folch M, Tapias JC, Huertas E (2007). Wastewater reclamation systems in small communities. Water Sci Technol.

[31] Lin T, Chen W, Cai B (2014). The use of chlorine dioxide for the inactivation of copepod zooplankton in drinking water treatment. Environ Technol.

[32] Sorlini S, Gialdini F, Biasibetti M, Collivignarelli C (2014). Influence of drinking water treatments on chlorine dioxide consumption and chlorite/chlorate formation. Water Res.

[33] Lénès D, Deboosere N, Ménard-Szczebara F, Jossent J (2010). Assessment of the removal and inactivation of influenza viruses H5N1 and H1N1 by drinking water treatment. Water Res.

[34] Wondergem E, van Dijk-Looijaard AM (1991). Chlorine dioxide as a post-disinfectant for Dutch drinking water. Sci Total Environ.

[35] Wen G, Xu X, Huang T, Zhu H, Ma J (2017). Inactivation of three genera of dominant fungal spores in groundwater using chlorine dioxide: Effectiveness, influencing factors, and mechanisms. Water Res.

[36] Casini B, Buzzigoli A, Cristina ML, Spagnolo AM (2014). Long-term effects of hospital water network disinfection on Legionella and other waterborne bacteria in an Italian university hospital. Infect Control Hosp Epidemiol.

[37] Chen YQ, Chen C, Zhang XJ, Zheng Q, Liu YY (2012). Inactivation of resistant Mycobacteria mucogenicum in water: chlorine resistance and mechanism analysis. Biomedical and Environmental Sciences.

[38] Heiner JD, Simmons EA, Hile DC, Wedmore IS (2011). A blinded, randomized, palatability study comparing variations of 2 popular field water disinfection tablets. Wilderness Environ Med.

[39] Liu GH, Du Lin (2011). Study on application of chlorine dioxide as disinfectant in water treatment. International Conference on Remote Sensing, Environment and Transportation Engineering.

[40] Barbeau B, Desjardins R, Mysore C, Prévost M (2005). Impacts of water quality on chlorine and chlorine dioxide efficacy in natural waters. Water Res.

[41] Chauret C, Volk C, Stover L, Dykstra T (2005). Effect of disinfectants on microbial ecology in model distribution systems. Journal of water and health.

[42] Huber MM, Korhonen S, Ternes TA, von Gunten U (2005). Oxidation of pharmaceuticals during water treatment with chlorine dioxide. Water Res.

